# O12.3. PROTECTIVE FACTORS FOR PSYCHOTIC EXPERIENCES AMONGST ADOLESCENTS EXPOSED TO MULTIPLE FORMS OF VICTIMIZATION

**DOI:** 10.1093/schbul/sby015.271

**Published:** 2018-04-01

**Authors:** Eloise Crush, Louise Arseneault, Terrie Moffitt, Andrea Danese, Avshalom Caspi, Sara Jaffee, Helen Fisher

**Affiliations:** 1 King’s College London, Institute of Psychiatry; 2 King’s College London; 3 King’s College London, Duke University; 4 University of Pennsylvania

## Abstract

**Background:**

Experiencing multiple types of victimization (poly-victimization) during adolescence is associated with onset of psychotic experiences. However, many poly-victimized adolescents will not develop such subclinical phenomena and the factors that protect them are unknown. This study investigated whether individual, family, or community-level characteristics were associated with an absence of psychotic experiences amongst poly-victimized adolescents.

**Methods:**

Participants were from the Environmental Risk (E-Risk) Longitudinal Twin Study, a nationally-representative cohort of 2232 UK-born twins. Exposure to seven different types of victimization between ages 12–18 was ascertained using a modified Juvenile Victimization Questionnaire at age 18. Adolescents were also interviewed about psychotic experiences at age 18. Protective factors were measured at ages 12 and 18.

**Results:**

Exposure to poly-victimization during adolescence was associated with age-18 psychotic experiences (OR=4.62, 95% CI 3.59–5.94, P<0.001), but more than a third of the poly-victimized adolescents reported having no psychotic experiences (40.1%). Greater social support was found to be protective against adolescent psychotic experiences amongst those exposed to poly-victimization. Notably, social support was also generally associated with a reduced likelihood of age-18 psychotic experiences in the whole sample (along with engaging in physical activity and greater neighborhood social cohesion).

**Discussion:**

Increasing social support from friends and family appears to be an important area for preventive interventions targeting adolescent psychotic experiences. Such prevention efforts would be most effectively targeted at poly-victimized adolescents who are at high-risk of developing psychotic phenomena.

